# University sports in Lebanon: between French heritage and American influence

**DOI:** 10.3389/fspor.2026.1829433

**Published:** 2026-08-18

**Authors:** Denis Bernardeau-Moreau, Nadim Nassif, Elie El Hayek

**Affiliations:** 1 Univ. Lille, ULR 7369—URePSSS—Unité de Recherche Pluridisciplinaire Sport Santé Société, Lille, France; 2 Department of Psychology, Education, and Physical Education, Notre Dame University–Louaize (NDU), Zouk Mosbeh, Lebanon; 3 Independent Researcher, Zouk Mosbeh, Lebanon

**Keywords:** American sports model, French sports model, Lebanon, students' sports scholarships, university sports

## Abstract

**Introduction:**

This article examines the structure of university sports in Lebanon, a system that remains mostly overlooked in academic research.

**Methods:**

By analyzing the governance of the Lebanese University Sport Federation (FSUL) through data collected from 131 student-athletes across 24 universities and interviews with 22 sports directors, this study identifies a particular model mixing variables from the French and North American sports models.

**Results:**

While the administrative rules are based on French traditions, the growing use of students' sports scholarships shows a visible shift toward the American collegiate system. The results also show that universities are not yet the primary source for training top athletes; instead, sports associations still lead athletes' development.

**Discussion:**

The study concludes that Lebanon is currently caught between the French and American university sports models while trying to build its own identity.

## 1 Introduction

The 19th and 20th centuries saw a crucial divergence in how sports developed on either side of the Atlantic. The North American sports model is characterized by a professional core that operates under a framework of revenue optimization and commercial expansion. Beneath this corporate layer, the responsibility for talent incubation and the governance of amateur play is largely delegated to the educational system (1). Consequently, the continuity of the American amateur pipeline is fundamentally sustained by the infrastructure of high schools and universities (2). This institutional synergy effectively transforms the U.S. education system into a fundamental catalyst for professional talent development. Conversely, the European sports framework is built upon a strong commitment to grassroots participation and the spirit of volunteerism (1). This system functions as an expansive pyramid, where a vast network comprising hundreds of thousands of amateur clubs and millions of community members provides the essential foundation for selecting professional sporting elites (3). Lebanon's strategic placement at the intersection of East and West has made it highly open to cultural and linguistic diversity, a trait reflected in its educational development.

The absence of centralized state coordination in Lebanon's fragmented landscape demands a closer look at how individual universities oversee their own athletic programs. Consequently, this article explores the specificities of the Lebanese university sports system, using the North American and French models as comparative points of reference. By determining where Lebanon stands between these two international standards, this research evaluates how such influences have contributed to the progression of university athletics and the nation's broader sporting identity.

The Lebanese university sports system—shaped by a dual influence from Western powers—is examined here through three primary research questions. These research questions provide the necessary structure to evaluate the sector's current standing and its future potential. First, this study investigates how the Lebanese university sports system is structured, aiming to describe the organizational structure of sports in Lebanon, in particular the roles and modalities of governance and the legal framework of the Lebanese University Sports Federation (FSUL- “Fédération Sportive Universitaire du Liban”). By establishing a clear empirical map of the local framework, this question provides the essential baseline required for a broader comparative analysis. Building on this, the research identifies attributes that distinguish the North American and French sports models, establishing theoretical benchmarks by outlining the structural and cultural differences between these two dominant international systems. By highlighting the specific characteristics and points of convergence of the North American and French models, the research creates a comparative perspective through which the Lebanese system can be accurately evaluated. Ultimately, the study addresses which international paradigm—North American or French—demonstrates a higher degree of alignment with Lebanese university sports. As the primary focus of the study, this question evaluates how Lebanon's sports structure corresponds with or diverges from established global models. The comparison serves a dual purpose: it identifies the inherent strengths of the Lebanese system while highlighting specific institutional components that could be adapted for reform. Ultimately, this evaluation offers the empirical data insights necessary to guide the strategic advancement and modernization of the Lebanese university sports environment.

To grasp this complex landscape, the second part of the paper begins by contextualizing the study, looking at the historical roots of the FSUL and how Lebanon's dual educational heritage shaped the current system. The third part details our mixed-methods approach and the data gathering process involving student-athletes and sports directors. In the fourth part, we present our findings, contrasting the French and American models to identify exactly where Lebanon fits on the spectrum. Finally, the last part closes the study with a comparative analysis of national collegiate sporting models.

## 2. Context of the study

### 2.1 The role of the FSUL in governance strategies

University athletics in Lebanon is centrally regulated by the FSUL, an organization which origins can be traced back to its founding in 1947. It was officially recognized by the Lebanese government in 1949 via the Presidential Decree No. 14064, which labeled it as a public utility, a status that gave it the mandate to develop sports at the national level. The FSUL has always maintained an international presence within the International University Sports Federation (FISU—“Fédération Internationale du Sport Universitaire”) since 1949.

While the federation's administrative lineage has undergone various shifts, it currently operates under the Lebanese Ministry of Youth and Sports (LMYS). The landscape of university sports in Lebanon cannot be understood without acknowledging how political dynamics deeply influence the Lebanese sport community. A pivotal shift took place in March 2007, when Decree No. 213 shifted the FSUL from the LMYS to the Ministry of Education. This move was far from a technical adjustment; it triggered a vigorous debate that mirrored the power struggles between rival political blocs. As Nassif (4) highlights, sporting dominance frequently acts as a conduit for political overrepresentation. Early groups such as the Kata’ib' and “Najjadeh” successfully leveraged sports to resist the French Mandate during the 1930s and 1940s before transitioning into political parties (5). Reiche (6) argues that instead of diffusing divides, sports often reinforce them. The country's confessional structure creates an environment that limits competition to factional silos, turning major sports like football and basketball into stages for political and sectarian rivalry.

These political tensions were also present in the 2021 elections, which saw significant maneuvering for control over the federation. According to S. Garabedian (personal communication, 2024), former president of the FSUL, these contests involved coordinated efforts by specific factions to ensure that political parties secure a strong foothold within the FSUL leadership.

### 2.2 The dual educational heritage

This governance structure sits atop an educational foundation rooted by foreign missionary influence. The establishment of the American University of Beirut (AUB), formerly the Syrian Protestant College and Saint Joseph University (USJ, Université Saint-Joseph) played a pivotal role in shaping Lebanon's dual-language landscape (7). More than mere academic hubs, these institutions acted as socio-cultural anchors, using their resources to strengthen ties between specific religious groups and social classes through targeted linguistic training (7).

## 3. Research hypotheses

Based on this context, this study posits three core hypotheses regarding the structure and function of university sports in Lebanon:

- **Hypothesis 1 (H1):** The Lebanese university sports mechanism functions as a reflective microcosm of the nation's broader sociopolitical and sectarian landscape. Beyond mere recreation, athletic platforms serve as performative arenas where political tensions and interest group identities are manifested. As Al-Masri (8) suggests, sports venues in Lebanon have transformed into theaters for the display of power and wealth—a consequence of two decades of intersecting local and global pressures. Similarly, Boukhater (9) underscores the tight correlation between confessionalism and athletic passion. This is evidenced by the symbolic weight of major clubs like Hekmeh and Al Riyadi, whose fanbases largely mirror the Christian and Sunni Muslim demographics of their respective Beirut neighborhoods. Consequently, this hypothesis asserts that university sports, as a mirror of society, are fundamentally embedded within Lebanon's complex sectarian fabric.
- **Hypothesis 2 (H2):** The organizational configuration of Lebanese sports is a direct outcome of shifting power dynamics and the conflicting interests of key institutional actors. This framework suggests that the system is shaped not only by formal legislative mandates but also by the informal negotiations and interactions between primary stakeholders, including governing bodies like the FSUL, academic administrators, and external sponsors.
- **Hypothesis 3 (H3):** The Lebanese university sports system has evolved into a particular framework that synthesizes the structural, cultural and philosophical traditions of both the North American and French paradigms. This evolution is rooted in Lebanon's historical “educational pluralism” (7). Because the higher education landscape was founded upon dual Western influences, the resulting sports framework has forgone a uniform national consolidation. Instead, the North American model—defined by campus centric competition and athletic scholarships—operates in parallel with the French “federalist” tradition of voluntary administration and club-based structures. This mixture is viewed as an adaptive mechanism designed to compensate for the absence of a centralized national elite sports strategy.

## 4. Materials and methods

Our research uses a mixed-methods that combine, by triangulation, quantitative and qualitative methods. In the context of this study, the objective is to cross institutional and documentary data with participants lived experiences within the Lebanese university sports ecosystem. This approach facilitates a multi-dimensional assessment of the sector by combining complementary approaches that are exploratory and qualitative, verification and lexical analysis.

### 4.1 Exploratory approach and identification of typological characteristics

The first phase of the exploratory inquiry adopted structured survey instruments targeting two distinct populations: active student-athletes (*N* = 131) and a strategic group of institutional stakeholders (*N* *=* 22), including sports directors and university officials. By deploying these questionnaires, the study identified the cultural, institutional, and contextual variables that characterize the Lebanese university sports model.

When examining the students-athletes' cohort, the investigation concentrated on the operational realities of varsity participation, in particular investigating the criteria for scholarship acquisition, the adequacy of training logistics, and the availability of campus-based athletic facilities. Beyond these metrics, our survey evaluated subjective indicators such as satisfaction with coaching staff and the underlying motivations driving institutional selection. Concurrently, the survey administered to sports directors addressed the administrative and institutional architecture of the system. This included a detailed analysis of scholarship management protocols—focused on allocation tiers and governing authorities—as well as an examination of performance targets, and strategic integration of athletics within the broader institutional mission.

### 4.2 Lexical and contextual data analysis

The analytical phase was designed to synthesize these diverse data streams through both statistical and lexical processing. Results were subjected to descriptive statistical analysis to identify significant trends and frequencies across the university landscape. The purpose of this analytical phase is to better understand, through interviews and speeches, the representations and perceptions of athletes on the sport model in which they evolve.

To enhance the interpretive depth of the study, a qualitative lexical analysis was performed on open-ended responses from a sub-sample of student-athletes (*N* *=* 30). This group was purposively stratified to include ten representatives from each of the three primary academic models: The Lebanese (Public), American, and French system. This dataset was analyzed using IRaMuTeQ software, leveraging the R statistical framework to conduct Reinert's Descending Hierarchical Classification (DHC) and co-occurrence similarity analysis. This systematic mapping of the thematic structure allowed for a robust visualization of the primary concerns and discourses within the student-athlete community.

### 4.3 Interviews and consultations of verification

Complementing this textual analysis, the research incorporated a series of semi-structured interviews conducted between 2024 and 2025 with six key figures or gatekeepers in the Lebanese sports sector. As detailed in Table 1, these experts were purposively selected based on their professional roles in governance and national policy, ensuring a high-level perspective on the structural challenges facing the industry. By categorizing these qualitative insights according to specific professional functions, the study provides a nuanced understanding of the policy-making environment, effectively bridging the gap between national administrative frameworks and ground-level athletic execution. These confirmation interviews make it possible to verify the data and results obtained during the exploratory interviews and lexical analysis.

**Table 1:** Profile of qualitative interview participants (2024–2025).

Name	Institutional affiliation & tenure	Primary thematic contribution
S. Garabedian	Former President, FSUL (2021–2023)	Historical evolution of Lebanese university sports frameworks
M. Oueidat	Head of Sports Department, Ministry of Youth and Sports	Structural positioning of FSUL within the national sports hierarchy
Z. Saadeh	Current President, FSUL (2024-Present)	Operational mechanics and competitive landscape of FSUL tournaments
M. El Khoury	Head of Athletics (USJ); Secretary of National Teams Affairs (FSUL)	Analysis of the University Sports Conference “USC” competitions organized by various private Lebanese universities
J. Salameh	Olympic International Lecturer; Sports Figure	Governance structures of the Ministry of Youth and Sports and the 29 Lebanese sports federations that are members of the Lebanese Olympic Committee
W. Abdelnour	Senior Sports Journalist	Media visibility and reporting trends of FSUL athletics

To facilitate the qualitative inquiry, a semi-structured interview protocol was developed to investigate the structural and historical dimensions of the Lebanese athletic landscape. This instrument was designed to capture the detailed representations and perspectives on the evolution of university-level competition and the administrative hierarchy governing the LMYS. Furthermore, the protocol addressed the organizational complexities of the 29 sports federations currently affiliated with the Lebanese Olympic Committee (LOC), providing a comparative macro-level view of national sports governance.

Beyond the primary stakeholder interviews, the research incorporated focused consultations to provide a comparative and legislative context. In February 2025, a framework survey on volunteer and professional works within French sports federations was conducted, drawing on the expertise of Prof. Denis Bernardeau-Moreau. This consultation focused specifically on the dynamics of compensation and the management of human capital within athletic organizations.

## 5 Results

### 5.1 Students-Athletes demographics and athletic profiles

The quantitative analysis featured 131 students-athletes from 23 diverse universities affiliated to the FSUL. During the 2023/2024 academic cycle, these participants compete across 13 distinct athletic disciplines, out of the 15 recognized by the federation. The cohort was characterized by a modal predominant age of 20 years (27.5%), reflecting a traditional undergraduate demographic. Regarding athletic commitment, the most common training frequency involved bi-weekly sessions of 90 min each (26.35%), establishing a baseline for the intensity of the Lebanese varsity model.

Over three quarters of the surveyed population (77.86%) are recipients of athletic scholarships, indicating that financial incentives are prevalent in the university sports landscape. The data indicates that institutional selection and initial award of the sports scholarships are primarily driven by technical proficiency (27.77%) and being affiliated with elite national clubs (21.29%). Once awarded, the retention of these scholarships is perceived by the athletes to be contingent upon consistent dedication and commitment to their respective programs. Interestingly, the presence of a professional athletic board and a high-caliber coaching staff was a decisive factor for nearly a quarter of the participants (22.9%) when selecting their university.

In terms of physical infrastructure, 80.91% of participants' report that their campuses are equipped with dedicated sports facilities. This availability of resources aligns with a high satisfaction rate (80.91%) regarding the quality of coaching and athletic services provided by their universities.

The final segment of the inquiry addressed the conceptual understanding of international athletic systems. The results suggest a notable gap in institutional knowledge, as 59.54% of student-athletes were unable to distinguish between the North American and French (European) athletic models. Among the minority who demonstrated familiarity (14.5%), the North American system was correctly identified as a closed competitive framework, while the European model was recognized for its hierarchical structure involving promotion and relegation mechanisms.

### 5.2 Lexical analysis of Lebanese student-athletes perceptions: an IRaMuTeQ-based inquiry

By utilizing IRaMuTeQ's suite of analytical functions—specifically the statistical, similarity, and Reinert (CHD) methods, alongside word cloud visualizations—we gain a detailed view of student-athletes' representations and perceptions concerning their sporting experience in Lebanon. A dominant thread runs through these diverse data points: the tendency of athletes to evaluate their local reality through a comparative lens, constantly measuring the Lebanese model against French and American standards.

In the eyes of the participants, the U.S. model represents the idealized model of opportunity, defined by robust scholarships, elite coaching, and high visibility. The French framework, meanwhile, is viewed primarily as a stable system of organization and structural support. Standing in stark opposition to these foreign ideals is the Lebanese system, which respondents frequently associate with logistical hurdles, inadequate infrastructure, and a general deficit of institutional backing.

Empirically, this dissatisfaction appears in the lexical data where the FSUL is statistically tied to descriptors like “weakness”. In contrast, terms such as “opportunity” and “compare” cluster tightly around references to “France” and “America”. Furthermore, the co-occurrence graphs reveal that “university” and “athlete” occupy central nodes, a proximity that highlights the participants' dual identity and emphasizes how critical they believe higher education institutions are to their athletic trajectories. Ultimately, the discourse reflects a binary vision: local universities are pitted against their Western counterparts—especially the American model—in terms of funding, facilities, and professional pathways. This analysis confirms that the perceived deficits of the system are not just theoretical; they are a lived reality for student-athletes who actively benchmark their own status against international archetypes.

### 5.3 Stakeholder perspectives on university athletic management

Data from the stakeholder cohort (*N* = 22) indicates a high degree of institutional investment in athletic incentives, with 81.81% of universities providing dedicated sports scholarships. The distribution of these awards is predominantly merit-based, with athletic proficiency and affiliation with elite national clubs cited as the primary criteria (42.85%). Financial coverage remains highly varied; notably, 16.66% of institutions offer sports scholarships between 10% to 20% while an equal percentage provides a broader range of 10% to 50%. Governance of these financial instruments is divided: 27.27% of scholarships are managed directly by athletic departments, while 22.72% fall under the jurisdiction of general university administrations.

Regarding fiscal infrastructure, 86.36% of institutions maintain a dedicated athletic budget. However, a significant administrative nuance was identified: in 25% of these cases, the designated sports budget is separated from scholarship funding. This suggests that for a quarter of Lebanese universities, athletic scholarships are treated as tuition waivers or general financial aid rather than departmental operational costs.

The research reveals a push toward professionalization in the sector, with 63.63% of universities establishing specific performance targets for their coaching and directorial staff. This trend of accountability is mirrored in recruitment strategies, as 63.63% of institutions actively engage in high school scouting. The relationship between university athletics and the elite club circuit is particularly robust. Notably, 95.23% of the surveyed sports directors attract student-athletes in their universities who are simultaneously integrated into elite clubs, with 33.33% maintaining a rigorous five-day weekly training schedule, including club and university training. The influence of the club system is further underscored by the fact that 84.21% of these athletes receive professional financial compensation from their clubs, suggesting a dual-career model that bridges education and professional sport.

Infrastructure management reflects a pragmatic, mixed-resource model. While 23.8% of universities possess on-campus facilities, the majority rely on external leasing (28.57%) or a mixed approach (47.61%) utilizing both proprietary and rented venues. There is a widespread consensus (90%) among directors that scholarships are the key factor for the development of Lebanese university sports. However, the evaluation of current competitive standards is more varied, with only 22.22% rating the quality of university events as “very good”. Crucially, 100% of stakeholders identified a need for structural enhancements. These recommendations focus on the administrative modernization of the FSUL (26.66%), the introduction of live broadcasting (20%), and more stringent regulatory oversight regarding officiating and rule consistency.

To establish a clear definition of the current Lebanese university sports model, Table 2 synthesizes the core operational and athletic variables identified through the dual-survey approach.

**Table 2:** Structural profile of the Lebanese university sports model: A multi-stakeholder synthesis.

Systemic variable	Administrators perspectives (*N* *=* 22)	Students-athletes perspectives *(N* *=* 131)
Sports Scholarships	81.81% of institutions provide scholarships	Scholarships are primarily driven by technical skill (27.77%) and varsity team selection (21.29%).
Training Frequency	45% offer 2 training sessions weekly; 95.23% of universities enroll student-athletes that train with elite clubs while 33.33% of them train 5 days a week.	26.35% of respondents practice twice a week (90-minute per session).
Infrastructure & Management	47.61% of institutions utilize a mixed (owned/leased) facility model; 63.63% set performance targets for coaches.	80.91% report high satisfaction with coaching quality.
Competition & Recruitment	100% advocate for improvements in university competitions; 63.63% employ active high school scouting.	N/A

**Table 3:** Comparative analysis of collegiate sporting models.

Variables	Lebanese model in universities	American model in universities	French model in universities
Financial Capacity	$5k–$100k	$152M–$252M	Not related to university teams
Pathways & Eligibility	Club-Varsity	Draft system	Club-Varsity
Governance of Collegiate Sport	FSUL	NCAA,NAIA, NJCAA	FFSU
Infrastructure in the universities	Mixed (Proprietary & Leased)	High-Performance Proprietary Facilities	Shared Institutional & Municipal Hubs
Media exposure	Emerging (MTV Lebanon)	Global/Commercialized	Minimal
Training Frequency	∼3 h Weekly	40–50 h Weekly (NCAA DI)	1 to 5 h Weekly
Athlete Support	Private Scholarships	NIL & Scholarships	Governmental support for education
University Sport Orientation	Predominantly Amateur/Semi-Pro	Elite Professionalization	Recreational Amateur and Educational
Elite Sport System	Club-Centric and Presence of University Scholarships	High school and Collegiate Focus	Club-Based National System

## 6. Placement of the Lebanese universities sports model between the French and U.S. university sports models

### 6.1 An analysis of the French university sports model

The elite sport model in France is characterized by a structure that manage the coexistence of sporting and academic development within the higher education landscape. This dual career system allows athletes to pursue both high-level competition and academic degrees simultaneously without one sacrificing the other. As dictated by Decree No. 2002–1010 (dated July 18, 2002), these development routes utilize specialized facilities intended to help both established and promising athletes reach peak performance levels while preparing for professional athletic careers through structures such as “pôles Espoirs” and “pôles France” (10). Furthermore, Circular No. 1455, issued on October 6, 1987, outlines the regulations for admitting elite athletes into higher education, urging university deans and regional directors to actively support athletes in balancing their academic and athletic responsibilities through measures like extending degree durations, reducing mandatory lecture attendance, and rescheduling exam dates (10).

The role of universities in the sporting sector is heavily influenced by the French Federation of University Sports (FFSU), which emerged after a strategic shift in 1978 to separate university athletics from school sports, formerly unified under the Association du Sport Scolaire et Universitaire (ASSU). By the decree of March 13, 1986, the FFSU was granted the authority to organize and promote amateur athletic competitions for students across various higher education levels (11). Unlike most federations, the FFSU is primarily supervised by the Ministry of Higher Education, though the Ministry of Sports collaborates in setting and executing the federation's athletic objectives as outlined in article 16 of the revised Law of July 16, 1984. The expansion of athletic practices in recent years has led to the emergence of three distinct categories of physical and sports activities:

1. Competitive sports managed by the FFSU.
2. Elite-level performances prioritized by specific universities in collaboration with the FFSU's ongoing coordination.
3. Activités Physiques, Sportives et Artistiques (APSA) which encompass all other forms of movement intended to address physical maintenance, general wellness, and public health rather than focusing on competitive outcomes (11).

The success of this framework is particularly evident in the arena of international competition, where results from the Athens Olympic Games rivaled those of Great Britain. According to research conducted by Bayle et al. (12), more than half of the 41 French medalists—21 in total— were students-athletes connected with the National Institute of Sport, Expertise and Performance (INSEP, “Institut National du Sport, de l'Expertise et de la Performance”). Founded in 1975 under the jurisdiction of the Ministry of Sport, this state-run institution is dedicated to helping elite athletes synchronize intensive training schedules with their academic pursuits. INSEP maintains the capacity to support up to 1,000 athletes, who are admitted based on the selection of their specific sports federations. To facilitate a superior educational environment, teachers conduct lessons directly at the athletic facilities, keeping class sizes limited to an average of 15 students to ensure that elite athletes receive personalized academic attention.

In this context, universities traditionally prioritize teaching, leaving the primary responsibility for sports training to sports associations (13). The competitions organized by the FFSU have therefore a minor importance in the French elite sport landscape. This is further evidenced by the lack of substantial media exposure of these events. Although the FFSU maintains a YouTube channel and employs social media to distribute content, these efforts are somewhat restricted and primarily target an internal audience.

### 6.2 An analysis of the U.S. collegiate sports model

The American sporting apparatus functions as a global outlier, characterized by a highly fragmented and highly professionalized governance architecture. Rather than following the centralized, state-led models prevalent in Europe, the U.S. system is sustained through four distinct pillars: the community sector, the scholastic/intercollegiate network, the Olympic/Paralympic movement, and the professional leagues. For Prakash et al. (14), the community sector serves as the foundation, focusing on grassroots initiatives, infrastructure expansion, and player development. The second sub-sector of USA sport is intercollegiate, composed of the National Association of Intercollegiate Athletics (NAIA), the National Junior College Athletics Association (NJCAA), and most notably, the National Collegiate Athletic Association (NCAA), which stands as the preeminent regulatory force in this space.

A defining hallmark of this model is its profound systemic autonomy. Unlike many nations, the U.S. does not maintain a federal Ministry of Sports. Consequently, the Olympic system—governed globally by the IOC—is managed domestically by the United States Olympic and Paralympic Committee (USOPC), which operates as a private entity free from government control. This culture of independence is even more pronounced in the professional sector, dominated by private leagues, such as the National Football League (NFL), Major League Baseball (MLB), National Basketball Association (NBA), National Hockey League (NHL), Women's National Basketball Association (WNBA), and Major League Soccer (MLS). In this environment, entrepreneurs and private organizations manage their franchises in alignment with U.S. business regulations rather than state mandates. This allows these organizations to establish their own internal rules and governance methods, a defining characteristic of the American “free-market” approach to athletics (14).

This autonomy permeates down even to specific sport organizations. As a case in point: USA Basketball utilizes a twelve-member board of directors to oversee a membership structure divided into professional, collegiate, scholastic, youth, and associate categories (14). This integration builds elite development directly into the academic journey, creating a “scholar-athlete” pipeline that accounts for nearly 80% of elite sport progression in non-winter disciplines (2). High school athletes must demonstrate exceptional performance to be recruited, particularly at the NCAA Division I level. In the pursuit of top-tier talent, universities often offer scholarships to gifted candidates whose academic markers—such as GPA and SAT scores—may deviate from the institutional average (15).

The financial scale of American college athletics reinforces its status as a major commercial industry. According to the NCAA's (16) financial statements, the organization generated a total revenue of $1,376,591,143 in 2024. Media and marketing rights fees remain the primary driver, contributing $948,383,496 to the total income. Approximately 60% of the NCAA's yearly income—roughly $600 million—is allocated directly to Division I member institutions and conferences. An additional $150 million is specifically dedicated to supporting Division I championships. In contrast, the redistribution to lower tiers is significantly smaller, with Divisions II and III receiving 4.37% and 3.18% of total NCAA revenue, respectively. These funds are primarily utilized to finance championship events and provide baseline operational support for their member institutions. When it comes to sports facilities, between 2009 and 2014, major Division I institutions allocated over $3.9 billion toward the construction and renovation for new athletic facilities (17).

The most transformative shift in recent history is the legal redefinition of the student-athlete. The 2021 onset of Name, Image, and Likeness (NIL) regulations fundamentally altered the dynamics of amateur sports. By allowing athletes to monetize their personal brands through endorsements, public appearances, and entrepreneurial efforts (18), this change ensures that elite athletes can accumulate significant resources long before they enter a professional league. In the summer of 2023, University of Texas quarterback Arch Manning reportedly secured $3.8 million in NIL contracts before playing his first collegiate down. Similarly, prominent basketball players star in nationwide commercials, volleyball squads enter into group agreements with global corporations, and football programs utilize donor-funded NIL collectives to attract prospective recruits. These dealings clearly demonstrate that college athletes have become business entities functioning within a billion-dollar sports market (19).

### 6.3 The Lebanese university sports model: between French heritage and American influence

The Lebanese sports terrain operates within a sphere distinctly removed from the highly decentralized, resource-rich American framework. While the latter thrives on institutional autonomy, the Lebanese setting is defined by a “weak state” reality (20) —a condition characterized by an inability to efficiently carry out core state functions. Lebanon assigns a small amount of financial funds to the sports sector, and the government does not possess a strategic roadmap to enable elite sporting excellence (20).

The impediments to athletic development in Lebanon are not merely fiscal; they are rooted in a broader sociopolitical context. Nassif (4) identifies a dual-layered corruption within the sector: financial misappropriation and administrative “parachuting”, where federation appointments are driven by partisan loyalty rather than meritocratic expertise. Furthermore, the pervasive influence of confessionalism creates a governance environment where political meddling is almost unavoidable. Historical data suggests that overrepresentation based on sect leads to a profound lack of transparency, making it difficult to foresee a top-down resolution to these systemic issues (21). Consequently, there remains a total absence of official government decrees or specific legislation regarding elite sports development (22).

The lack of a centralized state framework has forced the Lebanese sports sector to carve out a localized path. While a small segment of the student-athlete population benefits from scholarships, the vast majority of elite development occurs within sports clubs. This dynamic highlights the secondary role of universities; while they are viewed as assets that improve the athlete's journey, they have yet to achieve the crucial status that defines the U.S. collegiate model.

Currently, the Lebanese approach exists at a tricky intersection of European tradition and American-style innovation. The function of university sports in Lebanon remains restricted, largely mirroring the French model of governance as evidenced by the parallels between the FFSU and the FSUL. However, it would be a mistake to label the systems as identical. Unlike Lebanon, France possesses a solid national structure for elite sports that diminishes the need for university-level intervention. In Lebanon, the university system must operate without that state-level support, creating a distinct set of pressures and expectations.

There are increasing signs that this traditional role is undergoing a shift. A few select institutions, particularly those operating under American educational standards, have begun to roll out sports scholarships—a hallmark of the U.S. system that is generally absent from the French tradition. This synthesis of influences is meaningful; although these initiatives are currently limited to a few specific cases, they introduce a student-athlete focused dynamic that was previously missing. This evolution suggests that the Lebanese university sports field is gradually shifting toward a more modern, integrated framework that may eventually offer a more visible pathway for the nation's athletic talent.

The recent surge in commercial interest suggests that this mixed model is not merely a theoretical concept but a blossoming economic reality. The partnership involving MTV Lebanon, OMT (Online Money Transfer), and the FSUL serves as a definitive turning point for the visibility of university athletics. According to recent media reports (Personal communication with Wadih Abdelnour, 2025), the 2025 basketball and futsal championship finals benefitted from extensive corporate support and national media coverage, marking a departure from the historical unseen nature of collegiate sports in Lebanon. This level of professional broadcasting and sponsorship indicates that the private sector is beginning to fill the structural gap left by the central government. By leveraging corporate interest, the university system is effectively laying the groundwork for a resilient athletic framework that operates independently of traditional state-led models.

## 7. Discussion

### 7.1 Media promotion and national consciousness

The divergence in media promotion across France, Lebanon, and the United States acts as a deep-seated symptom of how deeply—or shallowly—university sports are embedded in the national consciousness. In France, an institutional singularity prevails. By delegating almost all coverage to the official FFSU platforms, mainstream giants like “L”Équipe” effectively treat university athletics as a niche educational byproduct rather than a legitimate journalistic pursuit. This creates a closed-loop ecosystem that stifles the cultural growth of the student-athlete, ensuring the audience remains confined to those already within the academic circle.

In contrast, in Lebanon, there is a raw attempt to bridge a massive infrastructure gap. While the FSUL lacks even a basic digital footprint or official website, its 2025 partnership with OMT to broadcast finals on MTV Lebanon represents a shrewd pivot toward the private sector. This move suggests that in the absence of state-led digital hubs, the Lebanese model is bypassing traditional institutional growth to seek immediate traction through legacy television.

This stands in stark opposition to the American NCAA, which operates as a global media juggernaut. The sheer scale of ESPN's reach—distributing the 2015 Men's Final Four to 179 countries and regions (23). This phenomenon demonstrates that U.S. college sports are no longer merely a “student experience”. Instead, they have become a primary export of American cultural leverage and economic power. Where the French model sees a student and the Lebanese model sees a local promotional opportunity, the American framework constructs a professional-grade commercial product with a worldwide presence.

In the final analysis, these different strategies do more than just record scores; they dictate the socio-economic value of the athlete. As broadcasting rights and digital engagement continue to mutate, the widening gap between these national frameworks will likely decide whether the “student-athlete” remains a local amateur or transforms into a global brand.

### 7.2 Athlete support systems: NIL vs. governmental students-athletes support

Beyond the superficial reach of the camera lens, the true infrastructure of university sports lies in how nations support their athletes' livelihoods and professional futures of their athletes. This comparison exposes a profound divergence between the American logic of commercial independence and the French model of state-led protectionism.

In the United States, the implementation of NIL regulations has radically altered the financial landscape of collegiate athletics. By allowing athletes to generate approximately $917 million in the first year alone—a figure that has since crested into a billion-dollar annual industry—the U.S. has effectively reshaped the student-athlete into an autonomous commercial entity (24).

In sharp contrast, the French system dismisses this market-driven autonomy in favor of a strictly institutional and educational framework. The governmental support status personifies this philosophy, offering academic flexibility and training assistance rather than direct financial compensation. Under this model, the student-athlete is not a brand but a ward of the state; they must be registered on a formal ministerial list and recognized by the Ministry of Sports and relevant federations to receive institutional support (25).

The practical application of this French model was striking during the 31st World University Summer Games, where the 5,000 competing athletes included a French contingent comprised entirely of students that are registered in this list. As defined by the ministry, this designation allows these individuals to mesh their higher education with the grueling demands of international competition, viewing high-level sport as a vehicle for national stature and the promotion of sporting values (26).

### 7.3 Training volume and athletic commitment

Beyond the reach of institutional support lies the daily reality of the student-athlete: the taxing hours spent on the pitch, in the gym, and in the film room. The level of commitment required ranges from a full-time professional workload to a casual extracurricular pursuit. In the United States, university programs demand a relentless level of dedication, essentially mirroring professional sports and solidifying the university's role as the primary engine for elite athlete development.

Data from the 2019 NCAA GOALS study highlights that student-athletes across all divisions dedicate a substantial portion of their week to athletics. Specifically, Division I athletes commit a median of roughly 33 h weekly, while Division II and III athletes follow closely with 31 and 28 h respectively. However, when factoring in the total athletic environment, the NCAA indicates that Division I athletes often exceed 40 to 50 h per week exclusively on their sport (27). This demanding investment—comparable at a full-time—is managed alongside the pressure of maintaining academic eligibility. A standard week for a Division I athlete encompasses 20–30 h of practice and conditioning, 10–15 h for competition and travel, and an additional 5–9 h for film and analysis and recovery protocols (28).

In sharp contrast to this rigorous American schedule, the French model operate at a noticeably different pace. In France, university training is largely a leisure-based activity. Unlike the regimented NCAA systems, French training schedules are governed by available resources and vary between one and five hours weekly. In this ecosystem, the university functions as a support hub, providing flexible schedules for elite athletes who mostly sharpen their skills within external private clubs rather than the campus itself.

Lebanon presents a middle ground where universities provide a vital competitive outlet in the absence of a structured national elite sports system. For many student-athletes, university sports represent a crucial opportunity often tied to scholarship incentives offered by private institutions. However, the physical burden remains modest compared to the U.S. model. According to sports directors within the FSUL, the majority of Lebanese student-athletes train only twice per week for approximately 90 min per session, amounting to a mere three hours of weekly preparation.

### 7.4 Financial capacity and budgets

While training frequency and media visibility define the public face of university athletics, the structural capacity of these programs is most clearly evident in their fiscal frameworks. The financial divide between these three national systems is not merely a matter of degree, but a fundamental gap in economic philosophy and scale, stretching from modest local funding to multi-billion dollar industries.

French university sports operate principally within a publicly-subsidized framework. Funding prioritize broad student engagement rather than university competitions. In stark contrast, the Lebanese landscape reflects a different fiscal reality. There is an investment in university varsity teams, with reported annual budgets typically fluctuating between $5,000 and $100,000 with some institutions omitting figures due to confidentiality. These figures are overshadowed by the American collegiate model, which exists in a unique economic realm. According to USA TODAY (29), top-tier American programs wield revenues and expenditures that match those of professional sports leagues. Premier athletic departments produce annual revenues between $152 million and $252 million—figures that place them on a global footing with elite European football clubs. To frame this scale, the media rights value of the Big Ten Conference now aligns with those of premier professional leagues such as Italy's Serie A or Germany's Bundesliga. Some American college athletic programs make as much money as top European football clubs (30). Ultimately, this financial chasm suggests that while the French and Lebanese models treat university sports as an institutional or developmental cost, the American framework has successfully transformed it into a high-yield global asset.

### 7.5 Synthesis of structural philosophies

The data presented in the comparative framework illustrates a profound divergence in financial capacity and institutional logic between the French, Lebanese, and American university systems. Rather than a single global standard for collegiate athletics, these findings uncover three distinct structural philosophies, each shaped by financial resources, institutional mandates, and the importance of sport in each nation.

The North American model operates within an aggressively commercialized environment, where university budgets and media footprints rival those of professional European sporting organizations. This professionalized reality has been further intensified by the landmark 2024 policy shifts regarding student-athlete compensation. In this context, the university is not merely an educational institution but the essential engine of the elite sport, serving as the primary feeder for both the Olympic movement and major professional leagues.

In contrast, the French system maintains a commitment to the recreational and pedagogical foundations of sport. The university is a site for educational development rather than professional scouting, as the elite routes to professionalization are historically and legally tethered to the national club system.

The Lebanese model emerges as a mixture form of these two extremes. While categorized as predominantly amateur, it demonstrates semi-professional characteristics through the intentional use of university scholarships. Within the Lebanese context, elite sport remains significantly less developed than in France and North America; nevertheless, the availability of university scholarship programs enables collegiate athletics to serve as a crucial motivational tool for talented athletes, potentially bolstering the overall quality of elite sports within the country.

## 8. Limitations

While this research provides a detailed mapping of the Lebanese university sports landscape, several limits must be acknowledged to contextualize the findings. The reliance on self-administered questionnaires and semi-structured interviews, while effective for capturing the lived experiences of student-athletes and directors, introduces certain natural compromises. Specifically, the absence of an intermediary during questionnaire completion means that nuances in participant interpretation could not be clarified in real-time—a common challenge in large-scale educational research.

The representativeness of the data was also shaped by varying levels of institutional engagement. Although the participation rate was remarkably high—with 24 of the 27 FSUL member universities contributing athlete data and 22 sports directors participating in interviews—the absence of the remaining institutions must be noted. While these gaps do not undermine the overall weight of the results, they do mean that certain localized institutional cultures or niche student-athlete experiences may be underrepresented. Future research would benefit from even deeper cross-institutional collaboration to capture these outlying perspectives.

The comparative ambition of this research was restricted by significant data hurdles, particularly regarding financial and operational metrics. Attempts to benchmark Lebanese university sports budgets and training frequencies against French counterparts were frequently met with confidentiality restrictions from SUAPS offices and Lebanese administrations alike. Thus, a thorough comparative examination of university sports budgets and training schedules across the three countries could not be fully accomplished.

Finally, this research has only compared the Lebanese university sport model to the French and American ones, due to the influence of both countries on the history of Lebanon. Although sociopolitical factors provide a useful context for interpreting these findings, the present exploratory study was not designed to directly examine these mechanisms. Further investigation should explicitly test the influence of these contextual variables. This also does not mean that the Lebanese model is not marked by other components that shaped its formation such as the Arab political context (Lebanon is a member of the Arab league) and the Asian sport environment (all the Lebanese sports federations are members of their Asian counterparts). These two variables must be examined deeply in future research to give a more holistic explanation on the governance of Lebanese university sport.

## 9. Conclusion

The findings of this research offer a significant contribution to the broader discourse on university sports systems, specifically by shedding light on the Lebanese context—a landscape that has remained seldom explored in global scholarly literature. By mapping institutional practices and governance, this study clarifies the mechanics of the Lebanese model, which is influenced by both the French and American structures.

The results suggest that while the Lebanese framework remains anchored in French traditions regarding its formal governance, the adoption of athletic scholarships signals a clear movement toward a new model that adopts sports strategies from U.S. colleges. This transition is a defining factor for the future of the FSUL. It shows that the Lebanese university sport system represents a specific configuration influenced by both French and North American institutional features.

A central takeaway from this analysis is that universities in Lebanon do not yet act as the main source for high-level athlete development. Instead, sports clubs continue to serve as the main venues for elite training and advancement. However, the expansion of scholarship programs is beginning to reposition university athletics as a crucial link for talented individuals, which may eventually enhance the overall quality of elite sports within the country. Ultimately, the Lebanese system—currently a blend of amateurism and emerging semi-professionalism—represents a compelling case of a sporting identity in the midst of a significant transformation.

## Data Availability

The raw data supporting the conclusions of this article will be made available by the authors, without undue reservation.
